# Intronomics-MIP: a snakemake pipeline for analyzing multilocus intron polymorphisms in species identification and population genomics

**DOI:** 10.1186/s13104-025-07264-6

**Published:** 2025-05-06

**Authors:** A. Scapolatiello, E. Boscari, L. Schiavon, N. Vitulo, L. Congiu

**Affiliations:** 1 Department of Biology, University of Padova, Via Ugo Bassi 58B, 35121 Padua, Italy; 2 Department of Biotechnology, University of Verona, Strada le Grazie, 15, 37134 Verona, Italy; 3 Consorzio Nazionale Interuniversitario Per le Scienze del Mare (CoNISMa), Piazzale Flaminio 9, 00196 Rome, Italy; 4 National Biodiversity Future Center, Palermo, Italy

**Keywords:** Bioinformatics pipeline, High-throughput DNA sequencing, Molecular markers, Multiple-SNP haplotypes, Non-model organisms, Teleost fishes

## Abstract

In this Research Note, we introduce Intronomics-MIP, a snakemake-based pipeline for the automated analysis of multi-locus intron polymorphisms (MIPs) using intron-targeted amplicon sequencing. Building on established methodologies, our pipeline integrates tools such as Cutadapt, FLASH, and SeekDeep to efficiently process and analyze highly variable intron regions. These MIPs serve as powerful multiple-allelic markers, primarily useful for distinguishing species, identifying cryptic species, disentangling species complexes and detecting hybridization, but can also be informative for assessing population structure without prior species knowledge. Our pipeline enhances reproducibility and scalability, making it adaptable to a wide range of taxa, with a specific demonstration on teleost species. We provide a comprehensive overview of the pipeline’s design, along with performance assessments using representative datasets.

## Introduction

The study of species groups, particularly those where interspecific hybridization or introgression is known or suspected, needs the analysis of shared bi-parentally inherited molecular markers. Traditional approaches, such as microsatellites [2, 4] or genome-wide SNP analyses [5, 16], have been widely used, but recent advancements have introduced intron-targeted amplicon sequencing as an effective alternative. This approach characterizes multi-locus intron polymorphisms (MIPs) [1], leveraging highly variable intron regions that are transferable across species.

MIPs have proven to be versatile nuclear markers, effectively distinguishing closely related species and populations with varying structures [1]. This method is especially advantageous for monitoring interspecific hybridization due to the hypervariable nature of these loci and the relatively long sequences they produce, facilitating the development of diagnostic markers. In particular, the approach has been successfully applied to teleost fish, a group characterized by high species diversity and complex evolutionary histories, as demonstrated in Boscari et al. [1].

Moreover, since the analysis of hypervariable intronic regions is anchored to the flanking exonic regions, which are selected for their high degree of conservation, MIPs offer the advantage of high transferability across species. The utility of MIPs extends beyond fish species, as this technique can be adapted for use in other taxa, revealing new sources of genetic variation.

To automate and streamline the analysis of MIPs, we developed a Snakemake-based pipeline [9] that integrates several bioinformatic tools into a cohesive workflow. This pipeline enhances reproducibility and enables scalable and efficient processing of large datasets.

To test the pipeline, we used the same samples reanalyzed the same samples from Boscari et al. [1], allowing for a direct comparison of the efficiency and speed of the scripts used to automate the workflow. This comparison provides a benchmark for evaluating the improvements introduced by our automated pipeline.

The pipeline output includes a table listing the allele names with their respective coverage, a Genepop file for population genetics analyses, and a folder containing the intronic sequences.

## Methods

### Overview of the pipeline

**Fig. 1:**
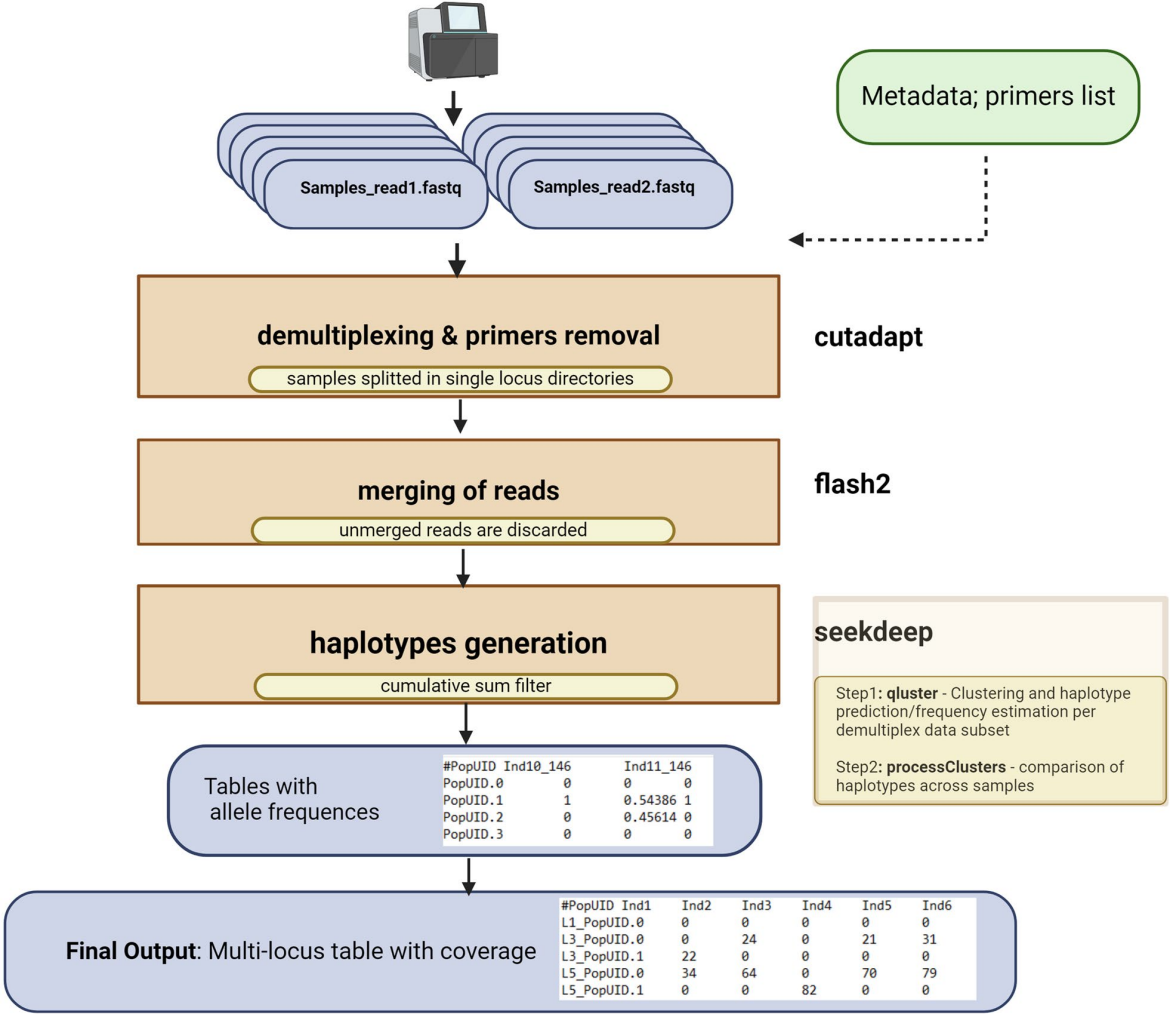
Flow diagram of the Intronomic-MIP pipeline from preprocessing to haplotype generation. Raw fastq reads processing: Cutadapt is used to demultiplex based on loci primers, discard low-quality bases, and remove adapters, setting a quality cutoff. Merging paired-end reads: FLASH merges pair-end reads, discarding loci where merged reads are < 10% of total. Reconstructing MIPs genotypes: SeekDeep pipeline clusters allelic variants, estimates allele frequencies with the qluster package, and filters out low-frequency alleles with processClusters. The final output retains only the top alleles by frequency for each locus and sample, with a cumulative fixed abundance

The pipeline is structured into sequential steps, or “rules” within the Snakemake python-based workflow manager, each corresponding to a specific bioinformatic process. The pipeline begins with the preprocessing of raw FASTQ files, followed by the merging of paired-end reads, and concludes with haplotype generation using SeekDeep [10]. A schematic overview of the pipeline’s workflow is provided in Fig. 1.

### Preprocessing of sequencing data

The first rule involves the script “split.py,” which is used as follows:
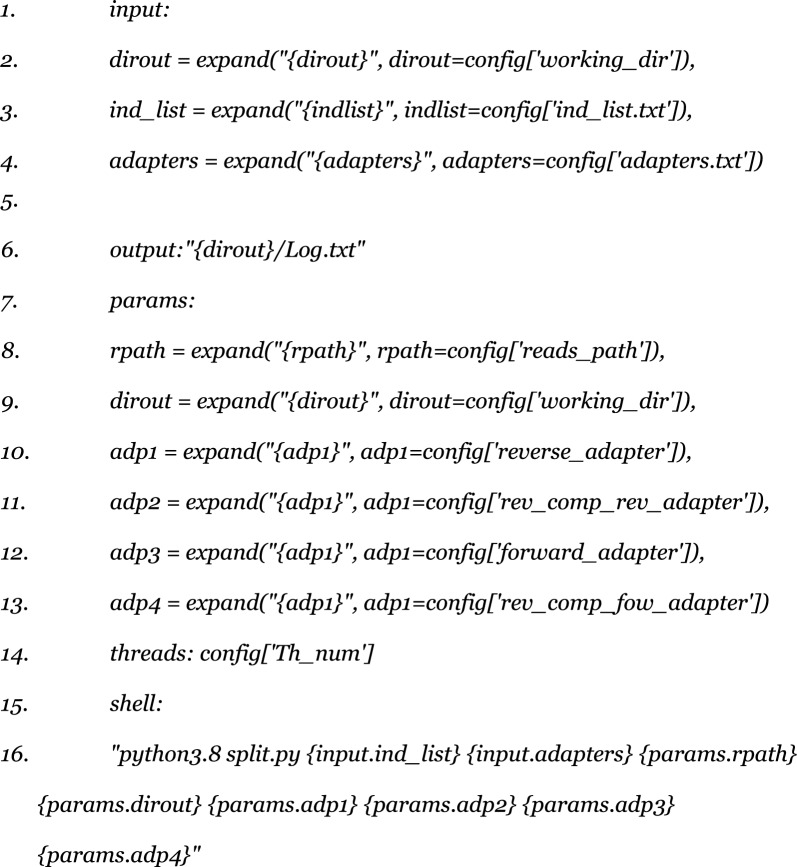


The input includes the list of samples, locus-specific primers, and the adapters used for sequencing (including reverse complements). This script is designed to automate the process of trimming and filtering raw FASTQ files using Cutadapt (v2.8) [8], ensuring that sequences are processed according to the specific adapter-locus information provided. The script handles multiple samples and outputs processed data in the specified directory structure.

The key parameters used are as follows:

- Quality cutoff (-q): 15. We chose a balance between maintaining good read quality and retaining a large number of reads, achieving an accuracy of 97%.
- Minimum read length after trimming: 100 bp. To avoid including poorly alignable and low-quality reads in the analysis.
- Minimum overlap length with primer sequence: 15 bp. A 15 bp overlap ensures good primer-target affinity without excluding potentially useful reads, given the primer length of 18–25 bp.

### Merging of paired-end reads

To simplify the management of output folders, a script was added that renames the samples numerically and sequentially (e.g., Ind1, Ind2…).

After the trimming step, paired-end reads are merged using the FLASH (v2.2.00) program [7], which identifies overlaps between the reads in each pair. FLASH uses a default minimum 10 bp overlap for merging reads. We set a maximum overlap of 300 bp to merge only reads with overlaps under 300 bp. Primers targeted intronic regions in the 300–600 bp range to generate comparable sequences. Sequences shorter than 300 bp are excluded because they can present challenges during gel electrophoresis, making them less suitable for subsequent construction of rapid identification kits. In our case intronic sequences longer than 590 bp are discarded because they do not allow the minimum 10 bases overlap of the paired reads, (sequencing length is 300 bp). The pipeline handles this with the following Snakemake rule:
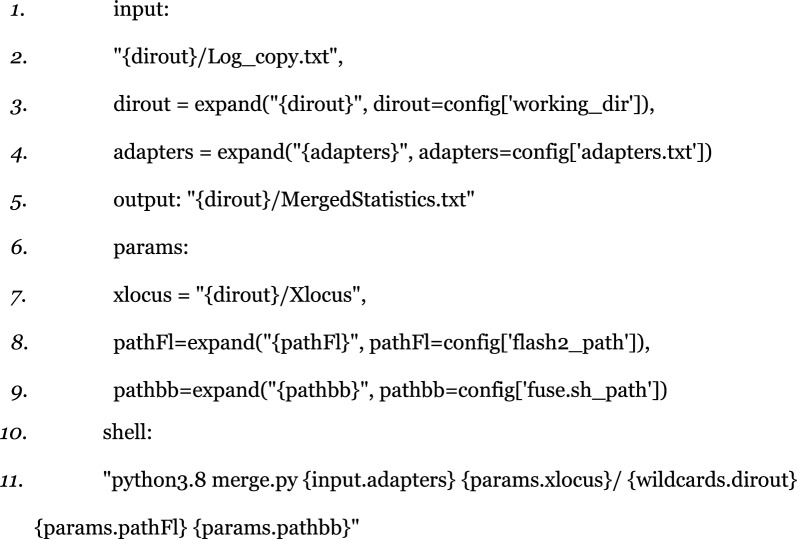


A different rule that uses a handmade script checks if the number of merged reads is less than 10% of the total reads for a given locus; in this case the locus is discarded, as this is insufficient to generate reliable haplotypes.

### Haplotype generation

An additional step involves using SeekDeep (v3.0.0) to perform de novo clustering of allelic variants. The algorithm assesses the genetic similarity between variants based on metrics such as distance matrices or sequence alignment scores. Variants are grouped into clusters where variants within the same cluster exhibit a high degree of similarity, indicating that they likely represent the same allele. In this step samples where the coverage is < 30 sequences are discarded*.* Post-clustering refinement steps may also be employed to reduce noise, merge closely related clusters, and eliminate outliers.

This step is crucial for accurately identifying and characterizing alleles across the samples, and it includes two main components:

- **qluster:** This component is responsible for predicting and estimating allele frequencies. To increase sensitivity to potential chimeras, we adjusted the command by adding the parameter –parFreqs = 1.5. The parFreqs parameter, which stands for Chimeric Parent Frequency multiplier cutoff, was originally set to a default value of 2. By lowering this value to 1.5, the pipeline becomes more sensitive in detecting and accounting for chimeric sequences, which is critical for ensuring the accuracy of haplotype reconstruction. Qluster uses quality scores to differentiate true variants from errors and k-mer frequencies to filter low-abundance PCR artifacts. The process starts by collapsing identical reads into clusters, indexed by k-mers and sorted by abundance. Clusters are iteratively compared and merged based on majority-rule consensus if thresholds are met. After each iteration, consensus sequences are updated and clusters re-evaluated until no changes remain (for more details, refer to Hathaway [10]).
- **processClusters:** This component is used for filtering and comparing alleles across samples. We employed several specific parameters to refine this process:
  –illumina: This option indicates that the data originate from Illumina sequencing, optimizing the analysis for this technology.
  –noErrors: This parameter ensures that only sequences with no detectable errors (such as incorrect mutations, insertions, or deletions) are retained, thereby improving the reliability of the allele calls.
  –sampleMinTotalReadCutOff = 30: This parameter sets a minimum threshold for the total read count for each sample, discarding any sample with fewer than 30 reads. This helps eliminate samples with insufficient sequence coverage, reducing the likelihood of low-frequency alleles that are more likely to be artifacts rather than true biological variants.

These adjustments ensure that the haplotype generation process is both sensitive and specific, resulting in more accurate and reliable outcomes across the analyzed samples.
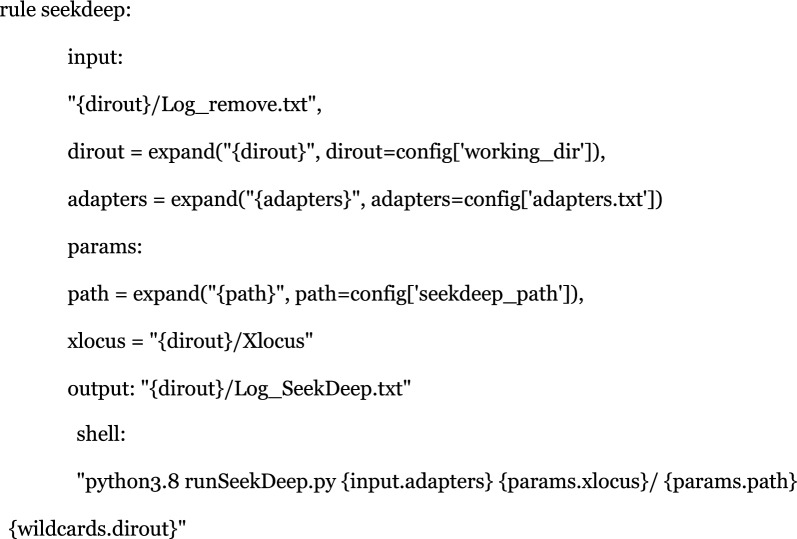


The final output of the workflow is a table that contains all alleles for each individual at every locus. Each allele is associated with a certain coverage, and thanks to the cumulativeSum parameter, adjustable in the configuration file (config.yaml), it is possible to discard probable low-coverage alleles (artifacts generated during amplification or sequencing) that escape SeekDeep’s filters. In this way, only alleles whose cumulative coverage exceeds the cumulativeSum % of total coverage are retained. Here’s an example:
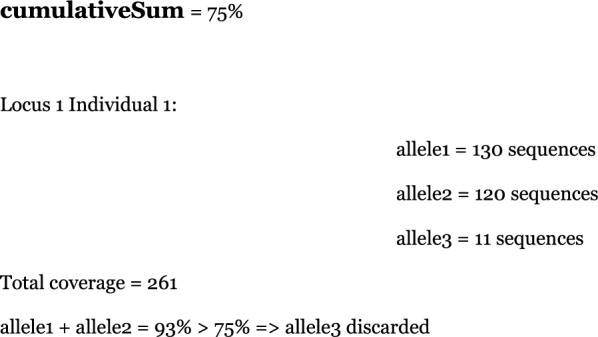


### Generation of a Genepop file

In addition to the final table containing genotype information and a folder with FASTA sequences of all alleles, the pipeline also provides a Genepop file [13] for further analysis, e.g. Structure. Alleles are coded by three digits (six digits for a diploid genotype, e.g., “001001” or “005081”), with missing data coded as “000000”. To test if the genotypes recovered from the pipeline contain the same biological information of the genotypes recovered in Boscari et al. [1], the Structure program (v2.3.4) [12] is used to infer the genetic structure of populations and to assign individuals to populations based on their genotypes.

## Results

The pipeline was tested on a dataset of 361 samples.

A total of 12.2 Gb of data was processed in 13 h, 7 of which were spent on the trimming step (working on a computer with AMD Ryzen 9 5900X 12-Core Processor 3.70 GHz and 32 GB of RAM).

**Fig. 2:**
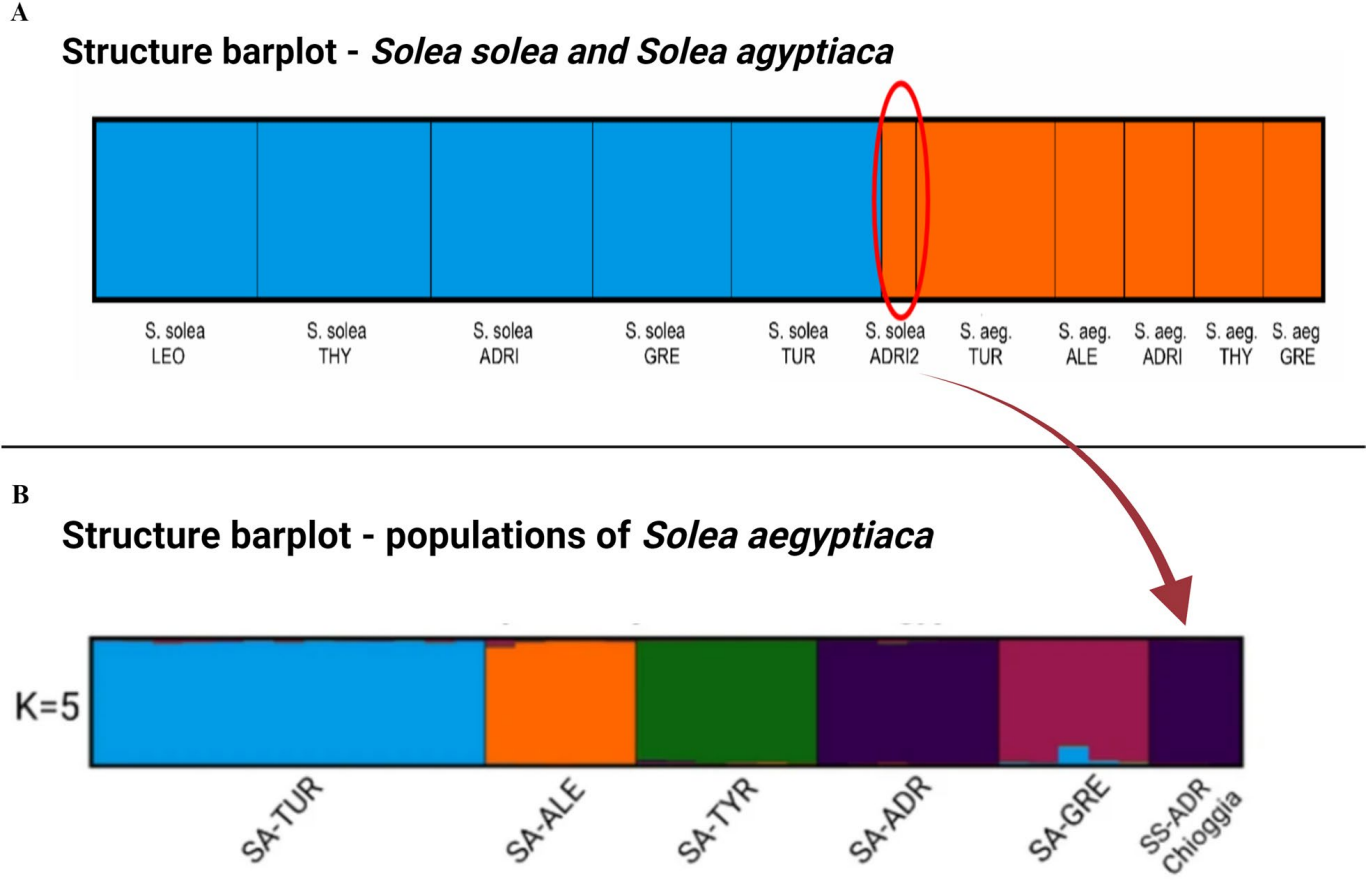
Comparison of the population structure graphs generated using Structure and post-processed with Clumpak [6]. **A** Membership probabilities of cluster assignment for the individuals of the two species. The analysis clearly shows that three individuals (highlighted by the red circle), which had been morphologically assigned to the species Solea solea, are in fact Solea aegyptiaca from the Adriatic Sea as demonstrated in the S. aegyptiaca population structure (**B**). [Solea solea = SS,Solea aegyptiaca = SA; Tyrrhenian Sea = TYR; North Adriatic Sea = ADR; Greece = GRE; Turkey = TUR; Alessandria = ALE]

To verify that our pipeline yields the same output given by manual procedure we reanalyzed the same 41 individuals of the species *Solea aegyptiaca* [14] previously analyzed by Boscari et al. [1], and used the output to perform Structure analyses (Fig. 2). The full overlapping of the results obtained by the two approaches, including the three individuals morphologically classified as *S. solea*, are genetically assigned to the species *S. aegyptica* (Fig. 2A) and to the population from the Adriatic Sea, with probability close to 100% (Fig. 2B).

## Discussion

The development of this Snakemake-based pipeline represents a significant advancement in automating the analysis of multi-locus intron polymorphisms (MIPs). By integrating established bioinformatic tools into a cohesive and automated workflow, this pipeline addresses key challenges in analyzing highly variable intron regions, particularly in terms of reproducibility and efficiency.

The identical population structure results coming from the pipeline and from the original methodology in Boscari et al. [1] confirm the robustness of the pipeline in handling complex datasets and producing reliable genetic insights.

A major strength of the pipeline is its ability to handle large and complex datasets. By automating multiple steps, from preprocessing raw data to generating haplotypes, the pipeline minimizes human error and ensures consistent and reproducible results across different datasets and research groups.

Moreover, the pipeline’s flexibility allows for its application across a wide range of taxa, extending beyond fish species. The ability to apply the same workflow to different organisms without extensive modification opens new opportunities for comparative studies and the discovery of novel genetic variation in non-model species [3]. This adaptability is particularly useful in ecological and evolutionary research, where universal markers like MIPs facilitate cross-species comparisons.

## Conclusions

This Snakemake-based pipeline provides a robust and scalable solution for the generation of haplotypes from raw multi-locus targeted sequencing data and represents a powerful tool for investigating genetic diversity, species differentiation, and hybridization. By automating the process, it ensures consistency and reproducibility, making it a valuable tool for researchers working with MIP markers and similar datasets.

### Limitations

While our pipeline represents a significant advancement in the automated analysis of MIPs, it has some limitations. The success of the pipeline depends on the quality of the input data. Despite the implementation of stringent filtering steps, low-quality reads can still lead to errors in haplotype generation, potentially skewing the results [11]. Even after trimming and filtering, low-quality reads may cause inaccurate haplotype calls, which can affect downstream analyses. Another limitation is that the pipeline’s effectiveness in detecting rare alleles might be constrained by the thresholds set for coverage filtering, potentially excluding biologically significant low-frequency alleles, especially in populations with high genetic diversity. Lastly, although the pipeline is generally robust, scalability may become a challenge when handling exceptionally large datasets or highly complex genomes [17, 18], where computational resources and processing time could become limiting factors.

A further issue concerns the use of references for haplotype generation. Although SeekDeep allows for the use of reference files, it does not easily manage output data. A future update of the pipeline could therefore integrate a rule for handling a priori information.

Additionally, we are exploring ways to automate the identification of species-specific alleles.

## Data Availability

The full code and documentation for the pipeline are available on GitHub: https://github.com/Nali-unipd/Intronomics-MIP.

## Requirements and code availability

The pipeline requires the following software:

 Snakemake v7.32.4

 Cutadapt v2.8

 FLASH v2.2.00

 SeekDeep v3.0.1

In the repository, there is an installation file designed to assist users in setting up the necessary prerequisites for the pipeline. Users can install the required software and dependencies using the following command:

 *bash install_dep.sh*

This script automates the installation process, ensuring that all necessary tools, including Snakemake, Cutadapt, FLASH, and SeekDeep, are properly installed on the user's system. Following the execution of this command, users will have all the prerequisites in place to run the Snakemake-based pipeline efficiently.

For any additional configuration or troubleshooting, please refer to the documentation provided in the repository.

The full code and documentation for the pipeline are available on GitHub [15].

A sample dataset and configuration file are also provided to help users get started.
